# Multifunctional cytomegalovirus (CMV)-specific CD8^+^ T cells are not restricted by telomere-related senescence in young or old adults

**DOI:** 10.1111/imm.12409

**Published:** 2015-03-09

**Authors:** Natalie E Riddell, Stephen J Griffiths, Laura Rivino, David C B King, Guo H Teo, Sian M Henson, Sara Cantisan, Rafael Solana, David M Kemeny, Paul A MacAry, Anis Larbi, Arne N Akbar

**Affiliations:** 1 Division of Infection and Immunity, University College LondonLondon, UK; 2 Immunology Programme and Department of Microbiology, National University of SingaporeSingapore; 3 Department of Immunology, Hospital Reina Sofia, University of CordobaCordoba, Spain; 4 Singapore Immunology Network, A*STARSingapore

**Keywords:** CD8^+^ T cells, cytomegalovirus, multi-functional, senescence, telomere

## Abstract

Antigen-specific multifunctional T cells that secrete interferon-*γ*, interleukin-2 and tumour necrosis factor-*α* simultaneously after activation are important for the control of many infections. It is unclear if these CD8^+^ T cells are at an early or late stage of differentiation and whether telomere erosion restricts their replicative capacity. We developed a multi-parameter flow cytometric method for investigating the relationship between differentiation (CD45RA and CD27 surface phenotype), function (cytokine production) and replicative capacity (telomere length) in individual cytomegalovirus (CMV) antigen-specific CD8^+^ T cells. This involves surface and intracellular cell staining coupled to fluorescence *in situ* hybridization to detect telomeres (flow-FISH). The end-stage/senescent CD8^+^ CD45RA^+^ CD27^−^ T-cell subset increases significantly during ageing and this is exaggerated in CMV immune-responsive subjects. However, these end-stage cells do not have the shortest telomeres, implicating additional non-telomere-related mechanisms in inducing their senescence. The telomere lengths in total and CMV (NLV)-specific CD8^+^ T cells in all four subsets defined by CD45RA and CD27 expression were significantly shorter in old compared with young individuals in both a Caucasian and an Asian cohort. Following stimulation by anti-CD3 or NLV peptide, similar proportions of triple-cytokine-producing cells are found in CD8^+^ T cells at all stages of differentiation in both age groups. Furthermore, these multi-functional cells had intermediate telomere lengths compared with cells producing only one or two cytokines after activation. Therefore, global and CMV (NLV)-specific CD8^+^ T cells that secrete interferon-*γ*, interleukin-2 and tumour necrosis factor-*α* are at an intermediate stage of differentiation and are not restricted by excessive telomere erosion.

## Introduction

The importance of T cells that have the ability to secrete interferon-*γ* (IFN-*γ*), interleukin-2 (IL-2) and tumour necrosis factor-*α* (TNF-*α*) simultaneously after activation in controlling HIV, hepatitis C virus and *Mycobacterium tuberculosis* infections in humans has been described previously.^1^–^5^ The maintenance of immunity during persistent or chronic antigenic challenge requires the continuous proliferation of antigen-specific T cells.^6^ Indeed, long-term non-progressing HIV patients demonstrate vigorous T-cell proliferative responses that are inversely correlated with viral load.^7^ Therefore, repeated episodes of proliferation and also the quality of the response in terms of cytokine production are both required for successful control of infections. One caveat is that continuous proliferation induces growth arrest or replicative senescence that is induced by the loss of telomeres.^8^ However, it is not known whether multifunctional CD8^+^ T cells have restricted proliferative capacity. The principal aim of this study was to investigate the relationship between cytokine production, cellular differentiation (determined by surface markers) and telomere erosion in individual cytomegalovirus (CMV) (NLV epitope)-specific cells.

Telomeres are repeating hexameric sequences of nucleotides at the ends of chromosomes that provide genomic stability but shorten with each cell replication.^9^ Eventually, a critically short length is reached and this induces growth arrest.^8^ Telomere erosion can be mitigated by induction of the enzyme telomerase in certain cells, which replenishes telomeric repeats at the ends of chromosomes and so extends proliferative lifespan. However, repeated antigenic stimulation of T cells results in loss of telomerase function, telomere erosion and replicative senescence.^10^ Previous studies have shown that CMV-specific CD4^+^ T cells have short telomeres when compared with Epstein–Barr, herpes simplex and varicella-zoster virus-specific populations in the same individuals and these cells have reduced capacity to proliferate in culture.^6^,^11^ This indicates that chronic CMV infection may restrict the proliferative capacity of T cells; however, it is not clear whether CMV-specific CD8^+^ T cells that have short telomeres also have restricted capacity to secrete cytokines.

Telomere length can be assessed by measuring telomere restriction fragments (TRF) after restriction enzyme digestion of DNA and by quantitative PCR (Q-FISH); however, these techniques are labour intensive, display variation between batches and require large amounts of DNA and previous subset isolation.^12^ Combining flow cytometry with fluorescence *in situ* hybridization (flow-FISH) provides a quick and reliable technique to analyse telomere length coupled with surface and intracellular parameters in different cell populations from a single small sample.^13^,^14^ We refined a flow-FISH technique that was described previously^6^,^15^,^16^ to investigate telomere length, surface phenotype and cytokine production in individual CD8^+^ T cells. We found that CMV-specific CD8^+^ T cells that secrete IFN-*γ*, IL-2 and TNF-*α* simultaneously are at an intermediate stage of differentiation as determined by surface phenotype and telomere length. Therefore, multi-functional CMV (NLV epitope)-specific CD8^+^ T cells are not restricted by replicative senescence.

## Materials and methods

### Blood sample collection and peripheral blood mononuclear cell isolation

Written informed consent was obtained and whole blood was collected in standard heparinized tubes from healthy volunteers. The study was approved by the Local Research Ethics Committee of the Royal Free and University College Medical School and the Singaporean National University Institutional Review Board. Donors did not have any co-morbidity, were not on any immunosuppressive drugs, and retained physical mobility and lifestyle independence. Peripheral blood mononuclear cells (PBMC) were isolated using Ficoll–Hypaque (Amersham Biosciences, Chalfont St Giles, Buckinghamshire, UK) and either analysed immediately or cyropreserved in 10% DMSO/fetal calf serum (FCS). Where stratified by age, young donors were between 18 and 40 years and old donors were 65 years of age and over.

### Determination of donor CMV status, CD8^+^ T-cell phenotyping, HLA-A2 typing and identification of tetramer-positive participants

The CMV status of donors was obtained by the overnight stimulation of fresh PBMC with CMV viral lysate and identification of IFN-*γ* production by CD4^+^ T cells [as described below; using CD4 conjugated with phycoerythrin-Cy7 (PE-Cy7), allophycocyanin (APC) or PE and IFN-*γ* conjugated with FITC or V450]. Previous data showed that there was total concordance between IFN-*γ*^+^ responses and seropositivity obtained from IgG serology obtained from the diagnostic laboratory of University College London Hospital. CD8^+^ T-cell phenotyping was achieved by incubating PBMC with CD8-peridinin chlorophyll protein (PerCP), CD8-FITC, CD27-FITC, CD27-PE, and CD45RA-APC (all from BD Biosciences, Oxford, UK). HLA typing was achieved by staining PBMC with HLA A2-PE (AbD Serotec, Kidlington, Oxfordshire, UK). Tetramer staining was conducted on HLA-matched CMV-responding donor PBMC for 20 min at 37° with HLA-A*0201 (NLVPMVATV) CMVpp65-specific tetramers, prior to antibody staining. Samples were acquired on a BD FACS Calibur of LSRII and analysed using Flowjo software.

### Global and NLV-specific T-cell stimulation

The PBMC were cultured overnight in RPMI-1640 medium supplemented with 10% FCS, 100 U/ml penicillin, 100 mg/ml streptomycin and 2 mm l-glutamine at 37° in a humidified 5% CO_2_ incubator. Cells were either left untreated or stimulated with 0·5 μg/ml of anti-CD3 antibody (purified OKT3) or 1 μg/ml NLV peptide (Proimmune, Oxford, UK) in the presence of recombinant human (rh) IL-2 (5 ng/ml) (R&D Systems, Abingdon, Oxfordshire, UK). After 2 hr, 5 μg/ml brefeldin A was added to each sample.

### Multi-colour flow-FISH analysis of global, MHC-class I tetramer-positive and cytokine-positive T-cell populations

We previously developed a modified version of the three-colour flow cytometry-FISH (flow-FISH)^15^,^17^ that used the heat-stable fluorochromes, Brilliant Violet- and Qdot nanocrystal-conjugated antibodies (BV and Q, respectively). This allowed simultaneous T-cell subset and antigen-specific or cytokine analysis in combination with telomere length measurement, so removing the need to pre-isolate cell populations. First, PBMC were either incubated with biotinylated -CD8, -CD28 or -CD45RA, or directly conjugated HLA-A*0201 restricted (NLV) CMVpp65 specific MHC-I tetramer before washing with PBS. Subsequently, cells were incubated for 15 min with streptavidin-conjugated Q800 or Cy3, fixable blue live–dead cell stain (Invitrogen, Inchinnan, Paisley, UK) and simultaneously stained with directly conjugated extracellular antibodies: FITC-CD8, -CD27 or -CD45RA; Q565-CD8; Q705-CD8; BV711-CD8, BV421-CD19; BV605-CD45RA; Q605-CD45RA; BD Horizon V500-CD27; BUV395-CD3 and BV786-CD27. For cytokine analysis, cell preparations were also fixed and permeabilized using Caltag Fix/perm buffers following the manufacturer's instructions. The intracellular antibodies (FITC-IL-2, BV605-IFN-*γ* and BV421-TNF-*α*) were added during the permeabilization step. Samples were then washed in PBS, fixed in 1 mm BS3 (30 min on ice, Thermo Scientific UK, Loughborough, UK) and quenched with 50 mm Tris–HCl in PBS (pH 7·2, 20 min, room temperature). Cells were then washed twice; first in PBS, and then in hybridization buffer (70% deionized formamide, 28·5 mm Tris–HCl pH 7.2, 1·4% BSA and 0·2m NaCl). Subsequently the samples were re-suspended in hybridization buffer and split between two or three new tubes (duplicates or replicates), incubated with 0·75 μg/ml of the PNA TelC-Cy5 probe (Panagene, Daejeon, South Korea) and heated for 10 min at 82°. Samples were then rapidly cooled on ice and left to hybridize for 1 hr at room temperature in the dark. Lastly, samples were washed twice in post-hybridization buffer (70% deionized formamide, 14·25 mm Tris–HCl pH 7.2, 0·14% BSA, 0·2m NaCl, 0·14% Tween-20) and twice in 2% BSA/PBS before acquisition on either the BD FACS Calibur or LSRII using cellquest software or BD FACS Diva software, respectively. Cryopreserved PBMC with known telomere fluorescence or Quantum™ Cy5™ Molecules of Equivalent Soluble Fluorochrome (MESF) beads (Bangs Laboratories, Fishers, Indianapolis, IN) were acquired alongside samples to ensure standardization of FACS machine set up. Qdots were purchased from Life Technologies (Paisley, UK), BVs from BioLegend (London, UK) or BD Biosciences and, unless stated otherwise, all other antibodies were purchased from BD Biosciences. All other reagents were purchased from Sigma-Aldrich (Dorset, UK) unless stated.

### Construction of the flow-FISH standard curve using Quantum™ Cy5™ MESF beads to determine telomere length

The Quantum™ Cy5™ MESF standard curve was constructed using nine samples stored in liquid nitrogen. These samples had known telomere length (as determined by TRF analysis) and were used previously to construct a standard curve based on median fluorescence intensity (MFI) of the telomere signal.^17^,^18^ Extracellular and intracellular Flow-FISH were performed, so that separate standard curves could be created for each procedure, and the samples were acquired on a BD LSRII simultaneously with Quantum™ Cy5™ MESF beads. The Quantum™ Cy5™ MESF beads standardize the Cy5 fluorescence of a sample by converting the MFI into a Molecules of Equivalent Soluble Fluorochrome unit (MESF) value. For both the extracellular and intracellular procedures, the MESF value for the telomere probe of each sample was plotted against the TRF kbp value and standard curves were generated using excel (Fig. 2e shows the extracellular MESF standard curve). In subsequent analysis the MFI of the telomere probe was converted into an MESF value and then into kbp using the constructed MESF standard curves. Use of the MESF standard curves allows accurate calculation of kbp from flow-FISH samples over time and between multiple instruments.

### Statistics

Statistical analysis was performed using either GraphPad Prism v6 (GraphPad Software, San Diego, CA) or IBM SPSS Statistics 20 (Armonk, NY). Data are presented as mean and *P *<* *0·05 was considered significant.

## Results

### CD45RA re-expressing T cells increase with age and/or CMV infection

**Figure 1:**
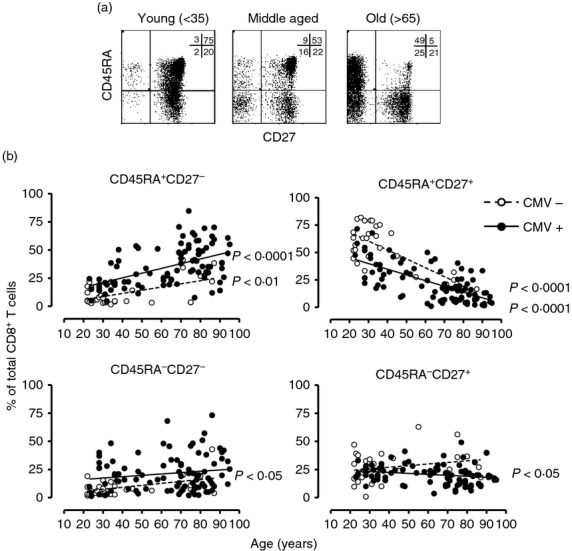
Composition of CD8^+^ T-cell compartment across the age range. Phenotypic analysis of CD45RA/CD27 expression on young, middle aged and old CD8^+^ T cells. Peripheral blood mononuclear cells stained for CD8, CD45RA and CD27 were analysed by flow cytometry. Representative pseudocolour plots for each age group are shown. Age in years is shown (a). Frequencies of each of the CD45RA/CD27 populations within total CD8^+^ T cells are represented in correlation to the age of the donors (b). Line of best fits for the cytomegalovirus (CMV) non-responders and CMV responders groups were generated by linear regression and the correlation assessed by Pearson and Spearman rank (GraphPad Prism).

CD8^+^ T cells can be divided into four subpopulations based on the expression of CD45RA and CD27.^19^,^20^ The frequency of each subpopulation as a percentage of the total CD8 compartment was recorded for all donors (Fig.1). In total, 125 healthy donors aged between 22 and 95 years were analysed, with 73% of participants demonstrating a CMV response, which is consistent with other reports.^21^ A line of best fit was generated for both the CMV responders and CMV non-responder groups by linear regression analysis and the correlation was assessed by Pearson and Spearman rank (GraphPad Prism) for each CD8^+^ T-cell subpopulation (Fig.1b). We found that the frequency of CD45RA^+^ CD27^+^ cells significantly decreased with age in both groups (Fig.1b; both *P *<* *0.0001), whereas the frequencies of CD45RA^−^ CD27^+^ T cells only decreased in CMV responders (*P *<* *0.05). CD45RA^−^ CD27^−^ T-cell frequencies were only found to increase with age in the CMV non-responding group (*P *<* *0.05), whereas both groups had greater CD45RA^+^ CD27^−^ T-cell frequencies with age (CMV responders; *P *<* *0.0001 and non-responders; *P *<* *0.01). However, this analysis does not take into account the individual contribution of ageing and CMV infection in the accumulation of CD8^+^ CD45RA^+^ CD27^−^ T cells.

To investigate this, data were further analysed by performing multiple linear regression analysis (see Supporting information, Table S1). A highly significant relationship existed between the decrease in CD45RA^+^ CD27^+^ T cells and increase in the CD45RA^+^ CD27^−^ T-cell population with either age or CMV response as independent factors (all values of *P* < 0·0001). These results are consistent with previous reports that used combination of different markers including CCR7 or CD11a and CD45RA^22^ or CD45RA and CD28^23^ to discriminate between the four cell subsets.

### Heat stability of quantum dot nanoparticle (Qdot), brilliant violet (BV) and brilliant ultra violet (BUV) fluorescent polymers relative to organic fluorochromes

**Figure 2:**
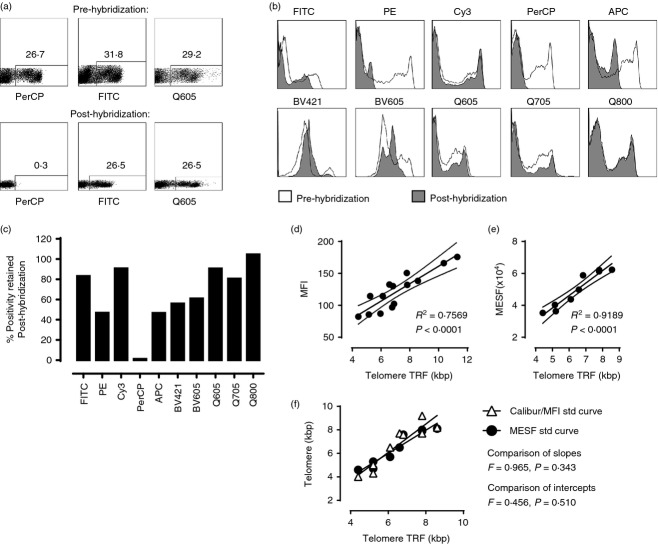
Fluorescent antibody stability and construction of MESF standard curve. Comparison of FACs plot analysis used to determine positive populations (a) and histograms depicting MFI (b) pre-hybridization (a, upper panel; b, open histogram) and post-hybridization (a, lower panel; b, closed histogram) for a selection of traditional organic fluorochromes versus Brilliant Violet (BV) and Qdot (Q) conjugated antibodies. Bar chart showing percentage of original signal retained post-hybridization (c). Representative examples of three experiments. Cryopreserved samples, which had a known telomere length as previously examined by Southern blot analysis of telomeric restriction fraction (TRF), were used to construct standard curves for future determination of telomere length of cells by Flow-FISH using either MFI on a BD FACS Calibur (d) or Cy5 MESF on a BD FACS LSRII (e). Lines of best fit and 95% CI were generated using linear regression and the correlations with TRF were assessed by Pearson and Spearman rank (Prism v6). The accuracy of the Flow-FISH techniques in measuring kbp was assessed by plotting the calculated telomere kbp of the nine participants used in both methods (MFI and MESF flow-FISH) compared with the actual kbp gained by TRF (f). The slope and *y* intercept of the two linear correlations (one for each method) generated by Prism were compared.

One of the main aims of the current work was to adapt the Flow-FISH technique to enable multiple parameter analysis of telomere length together with surface phenotype and cytokine production in small cell samples. As the detection of telomeres by this method requires heating of the sample to 82° for 10 min in hybridization solution, we first assessed the heat stability of antibody conjugates. As can be seen in Fig.2(a–c), the Qdots and BV conjugates tested all maintained a useable signal following the hybridization step compared with non-hybridized samples. The organic fluorochromes PE, PerCP and APC do not survive the treatment while FITC, Cy3 and Cy5 are relatively resistant (data not shown). It is of note that some antibodies are less tolerant of the hybridization process regardless of the reporter dye-conjugate. For example, an anti-CD28 signal was only detectable by using biotinylated-anti-CD28 followed by Q800- or Cy3-streptavidin conjugate, and was not detected when using a FITC directly conjugated antibody.

### Construction of kbp standard curve using Quantum™ Cy5™ MESF beads

To calculate the telomere length in kbp when using multi-coloured flow-FISH, a standard curve was constructed on a BD LSRII using cyropreserved PBMC with known telomere lengths and Quantum™ Cy5™ MESF beads similar to previous work.^17^,^24^ The correlation between MESF value and telomere kbp (as determined by TRF) was *R*^2^ = 0.9189 (Fig.2d). The MESF standard curve correlation was greater than the previously used MFI standard curve, which was created on the FACS Calibur using control samples to standardize MFI and correlating kbp length to sample MFI (Fig.2e, *R*^2^ = 0.7569). Using the nine samples that had been used to set up both the Calibur/MFI and MESF standard curves, we tested the comparability of the two standard curves by comparing the linear correlation of the kbp as determined by TRF to those gained by the Calibur/MFI curve and the MESF curve (Fig.2f). Statistical analyses demonstrated that there was no difference between the slope or intercept of the two linear correlations (*F* = 0·456, *P* = 0·343 and *F* = 0·456, *P* = 0·510, respectively). Results gained using either the Calibur/MFI standard curve or MESF standard curve were therefore comparable. Hence, for all multi-parameter experiments (more than four parameters), the MFI of the Cy5 signal of a cell population was converted to an MESF value using the Quantum™ Cy5™ MESF beads software (QuickCal, Bangs Laboratories), and this MESF value was then inserted into the following equations taken from the MESF standard curves: extracellular protocol; kbp = (MESF+680)/7804 and intracellular protocol; kbp = (MESF + 1773)/7046. As the Quantum™ Cy5™ MESF beads standardize the telomere Cy5 signal across time and different machines, when using the Quantum™ Cy5™ MESF beads and 0·75 μg/ml of the PNA TelC-Cy5 probe per sample, these linear equations may be used by researchers in different laboratories to calculate telomere kbp.

### Senescent CD45RA^+^ CD27^−^ CD8^+^ T cells have relatively long telomeres

**Figure 3:**
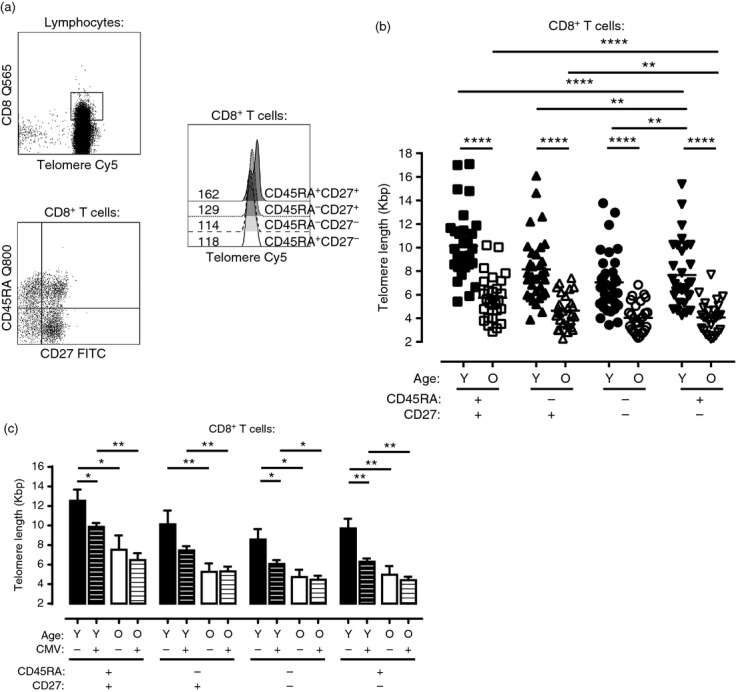
Telomere length of CD8^+^ T-cell subsets defined by CD45RA and CD27 expression within a mixed European and Asian cohort. Representative FACs plot analysis showing four-colour flow-FISH using biotinylated-CD45RA in combination with streptavidin-Q800, CD8-Q565 or V450, CD27-FITC and Cy5-PNA telomere probe (a). Hybridized CD8^+^ cells were gated from total lymphocytes before subset and telomere analysis. Values within histograms represent telomere MFI of the relevant populations. MFI was converted to kbp directly using the MFI/Calibur standard curve. Telomere MFI data collected on the Fortessa FACS Scan or LSRII alongside the Quantum™ Cy5™ MESF beads were first converted into MESF values before conversion into kbp using the MESF standard curve. Pooled data showing telomere lengths by age group of CD8^+^ CD45RA/CD27 (b: 32 young and 27 old donors). One dot represents one donor. Bar graph comparing telomere length of CD8^+^ CD45RA/CD27 T-cell populations segregated by both age and cytomegalovirus (CMV) status (c: *n* = 5–11). Y = young, O = old. Statistical analysis was performed using the two-tailed Student's *t*-test. Significant differences displayed by *values: *<0·05; **<0·01; and ****<0·0001.

Using a four-colour flow-FISH protocol, we first measured the telomere length in different CD8^+^ T-cell subsets that were defined using CD45RA and CD27 antibodies in a mixed Caucasian and Asian (majority Chinese or Indian Singaporeans) cohort. The Asian volunteers were recruited from and investigated in Singapore, whereas most of the Caucasian data were collected in London, UK. The samples were acquired on either a FACS Calibur alongside two control samples or a Fortessa FACS Scan or LSRII alongside the Quantum™ Cy5™ MESF beads to standardize the telomere signals. The FACS Calibur MFI was directly converted to kbp using the Calibur/MFI standard curve whereas the MESF telomere values were calculated using the QuickCal software and then converted to kbp using the MESF standard curve. CD45RA^+^ CD27^+^ cells had the longest telomere length in both young and old adults (Fig.3a, representative FACs plots and Fig.3b, cumulative data). The end-stage CD45RA^+^ CD27^−^ T-cell subset that exhibits multiple characteristics of senescent cells^25^ did not have the shortest telomere lengths, suggesting that telomere-independent mechanisms regulated the senescence in these cells. Telomere lengths in all four subsets were significantly shorter in the old compared with the young individuals (Fig.3b).

To determine whether different ethnicity had an influence on the observations, we investigated the Caucasian and Asian cohorts separately and compared telomere lengths within the CD45RA/CD27 CD8^+^ T-cell subsets (see Supporting information, Fig. S1). We found that the telomeres in the T cells of the Singapore cohort were shorter overall than in the samples from London (*P *< 0·05), nevertheless we observed the same trend in telomere lengths in both groups where CD45RA^+^ CD27^+^ cells had the longest while CD45RA^−^ CD27^−^ cells had the shortest telomeres (see Supporting information, Fig. S1a, b). Furthermore, the CD45RA^+^ CD27^−^ subset did not have the shortest telomeres in either cohort, suggesting the involvement of other mechanisms in inducing the senescence characteristics of these cells.^25^

We also investigated the telomere lengths in CD8^+^ T cells defined by their relative expression of CD28 and CD45RA (European cohort; see Supporting information, Fig. S1c, d). Similar results were obtained compared with using CD27 and CD45RA to distinguish between the cells; all the CD8^+^ T-cell subsets had shorter telomeres in the old compared with the young individuals and the CD45RA^+^ CD28^−^ population in both age groups did not have extremely short telomeres.

**Figure 4:**
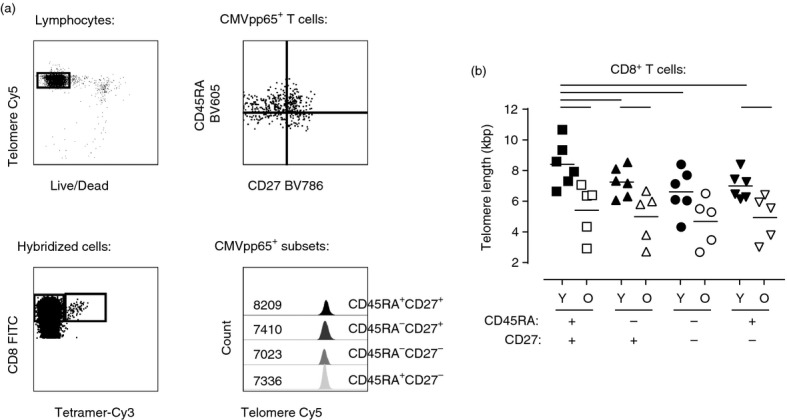
Telomere length of cytomegalovirus (CMV) pp65/HLA-A2 (NLV)-specific CD8^+^ T-cell subsets defined by CD45RA and CD27 expression. Representative example of six-colour flow-FISH to determine telomere length within NLV-specific CD45RA/CD27 subsets (a). Live hybridized cells from HLA-matched CMV responders were gated from total lymphocytes before gating of CD8^+^ tetramer^+^ cells. Subsequently CD45RA/CD27 subsets were gate from the tetramer^+^ population and the MFI of telomere probe for each population was determined. Data were acquired on a BD LSR II. Values within histograms represent telomere MFI of the relevant populations. Telomere length of NLV-specific CD45RA/CD27 CD8^+^ T-cell subsets stratified by age (b: 6 young and 5 old). Telomere MFI was converted first into an MESF value and then kbp using the MESF standard curve. One dot represents one donor, black dots represent young donors and white dots represent old donors. Statistical analysis was performed using the two-tailed Student *t*-test. All *P* values were < 0·05.

Cytomegalovirus seropositivity has an impact on the differentiation state of CD8^+^ T cells during ageing (Fig.1) and a previous report showed that chronic CMV infection also reduced the telomere length of the CD8^+^ T-cell pool.^26^ We therefore stratified young and old individuals on the basis of their CMV status and investigated telomere length in the four CD45RA/CD27 defined subsets. In young subjects, CMV infection induced a reduction of telomere length in each CD45RA/CD27 defined subset (Fig.3c). A similar trend was observed in old individuals but the difference in telomere length in the subsets between CMV-positive and CMV-negative subjects was not significant. We also investigated the telomere lengths in CMV (NLV)-specific CD8^+^ T cells in young and old individuals (Fig.4). The telomere lengths of these cells were shorter in old compared with young individuals and this difference was observed in each of the respective CD45RA/CD27 defined subsets (Fig.4b). The telomere length of each NLV-specific T-cell subset was also significantly shorter than the corresponding global CD8^+^ T-cell subset (data not shown). The CD45RA^+^ CD27^+^ NLV-specific CD8^+^ T cells had very short telomeres, indicating that these cells had undergone extensive proliferative activity *in vivo* and that these cells cannot be considered to be a naive population.

### Multi-cytokine-producing CD8^+^ T cells have intermediate telomere lengths

**Figure 5:**
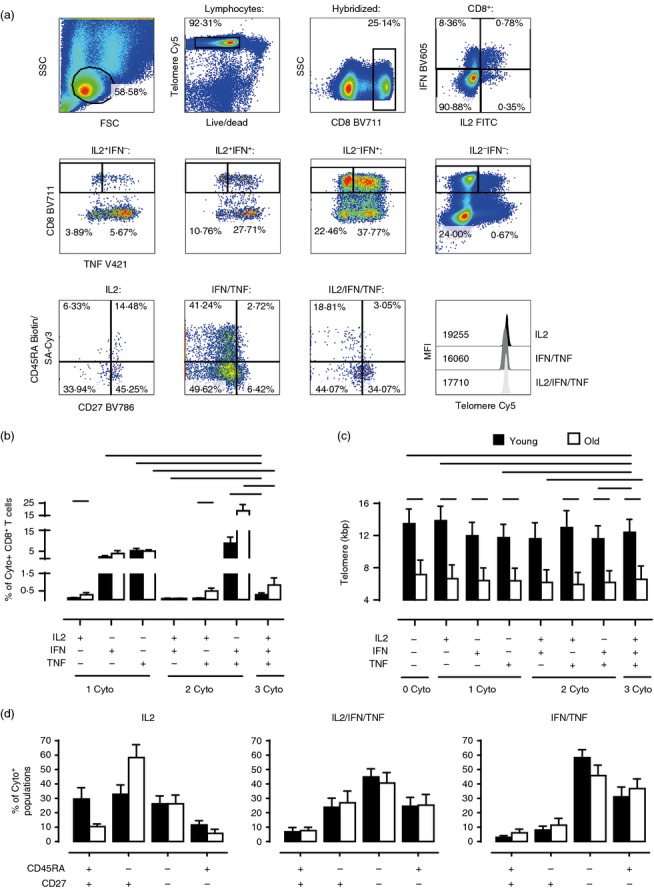
Simultaneous analysis of multiple cytokines, phenotype and telomere length within activated global CD8^+^ T cells. Total peripheral blood mononuclear cells (PBMC) were stimulated with plate-bound anti-CD3 and interleukin-2 (IL-2) overnight then assessed by eight-colour intracellular cytokine flow-FISH to determine telomere length and phenotype (CD45RA/CD27) of the different cytokine-producing populations [combinations of IL-2, interferon-*γ* (IFN-*γ*) and tumour necrosis factor-*α* (TNF-*α*)]. Representative example of gating strategy (a). First live hybridized cells were gated from lymphocytes, followed by gating of the CD8 population, and then IL-2/IFN-*γ* subsets (IL2^+^IFN^−^, IL2^+^IFN^+^, IL2^–^IFN^+^ and IL2^–^IFN^−^). Next a TNF-*α* positive and negative gate was placed on each of these four cytokine populations resulting in seven cytokine-positive populations: IL-2 only (IL2), IFN-*γ* only (IFN), TNF-*α* only (TNF), IL2+IFN+ (IL2/IFN), IL2+TNF*α*+ (IL2/TNF), IFN*γ*+TNF*α*+ (IFN/TNF) and IL2+IFN*γ*+TNF*α*+ (IL2/IFN/TNF). Finally, each population was assessed for CD45RA/CD27 expression and telomere MFI (Only data for the IL2, IFN/TNF and IL2/IFN/TNF populations are shown). Numbers in pseudocolour plots represent subset percentages; numbers in histogram represent telomere Cy5 MFI. Bar charts showing the size of each cytokine population as a proportion of total CD8^+^ T cells (b) and telomere length of each population (c) stratified by age (b, *n* = 6–8; c, *n* = 8). For simplicity, only significant differences between the two age groups and from the IL2/IFN/TNF population are shown (for full statistical analyses see Supporting information, Tables S2 and S3). Bar charts showing the CD45RA/CD27 subset distributions for the IL2, IL2/IFN/TNF and IFN/TNF populations (d, *n* = 5–8). Data were acquired on a BD LSR II. Telomere Cy5 MFIs were first converted to MESF values and subsequently kbp using the MESF standard curve. Black bars represent young, white bars represent old. Statistical analysis was performed using the two-tailed Student *t* test (SPSS v20). All *P* values were < 0·05.

We next investigated the relationship between telomere length, cell phenotype and capacity to secrete none, one or multiple cytokines simultaneously after CD3/IL-2 activation. To do this we measured telomere length in T cells stained for surface CD8/CD45RA/CD27 markers and intracellular accumulation of IL-2, IFN-*γ* and TNF-*α* after activation (Fig.5). Despite the fact that older individuals have significantly shorter telomere lengths within each cytokine-producing population compared with the young (Fig.5c, all *P *< 0.05 except the IL-2/IFN population, which generally contained very few cells), we found that the proportions of each cytokine-producing population responding to CD3/IL-2 stimulation were similar or higher in old individuals (Fig.5b). Of the cells that secreted cytokines, the populations that secreted IFN-*γ* alone, TNF-*α* alone or both cytokines simultaneously were the most abundant in both age groups (Fig.5b). There were a relatively low number of CD8^+^ T cells in young and old subjects that secreted all three cytokines. In both young and old individuals, the IL-2/IFN-*γ*/TNF-*α* multi-functional cells had intermediate telomere lengths relative to the other cytokine-producing cells within each age group, respectively (Fig.5c). We next investigated whether there was any relationship between the differentiation state of the cells defined by relative CD45RA and CD27 expression and their ability to secrete one or more cytokines. The cells that secreted only IL-2 in young and old subjects were found mainly in the less differentiated CD45RA^+^ CD27^+^ and CD45RA^−^ CD27^+^ populations (Fig.5d, left panel). Only a few CD8^+^ T cells secreted all three cytokines in the CD45RA^+^ CD27^+^ subset; however, these multifunctional cells were found in all of the other CD45RA/CD27 defined populations in both age groups (Fig.5d, middle panel). This, together with their relative telomere length, suggested that the IL-2/IFN-*γ*/TNF-*α* multi-functional cells were at an intermediate stage of differentiation. In contrast, cells that secreted IFN-*γ* and TNF-*α* simultaneously were mainly found in the highly differentiated CD45RA^−^ CD27^−^ and CD45RA^+^ CD27^−^ T-cell subsets (Fig.5d, right panel). Therefore, multifunctional cells are at an intermediate stage of differentiation and are present at equal frequency in both young and old subjects.

### Multi-cytokine producing CMV (NLV)-specific CD8^+^ T cells have intermediate telomere lengths

**Figure 6:**
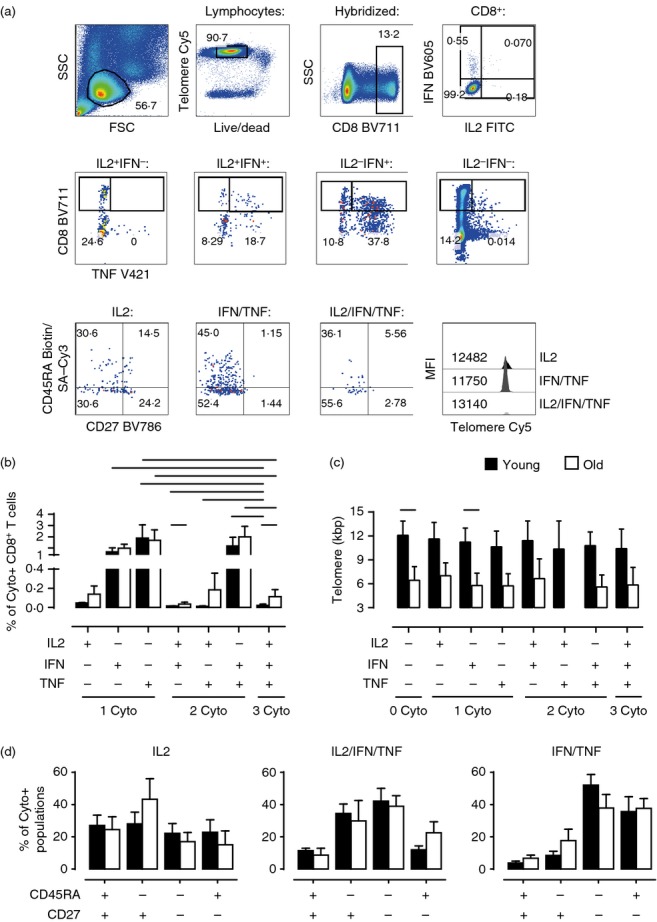
Simultaneous analysis of multiple cytokines, phenotype and telomere length within activated cytomegalovirus (CMV) pp65/HLA-A2 (NLV)-specific CD8^+^ T cells. Total peripheral blood mononuclear cells (PBMC) were stimulated with NLV peptide overnight then assessed by eight-colour intracellular cytokine flow-FISH to determine telomere length and phenotype (CD45RA/CD27) of the different cytokine-producing population [combinations of interleukin-2 (IL-2), interferon-*γ* (IFN-*γ*) and tumour necrosis factor-*α* (TNF-*α*)]. Representative example of gating strategy (a). For full explanation of gating see Fig.5. Numbers in pseudocolour plots represent subset percentages; numbers in histogram represent telomere Cy5 MFI. Bar charts showing the size of each cytokine population as a proportion of total CD8^+^ T cells (b) and telomere length of each population (c) stratified by age (b, *n* = 5–7 per group; c, *n* = 3–7 per group). For simplicity in (b), only significant differences between the two age groups and from the IL-2/IFN/TNF population are shown (for full statistical analysis see Supporting information, Tables S4 and S5). Bar charts showing the CD45RA/CD27 subset distributions for the IL-2, IL-2/IFN/TNF and IFN/TNF populations (d, *n* = 3–7). Data were acquired on a BD LSR II. Telomere Cy5 MFIs were first converted to MESF values and subsequently kbp using the MESF standard curve. Black bars represent young, white bars represent old. Statistical analysis was performed using the Wilcoxon Signed Rank test. All *P* values were < 0·05.

We next examined the cytokine production relative to telomere length within the CMV (NLV)-specific CD8^+^ T cells in young and old subjects (gating strategy in Fig.6a) and found that the observations paralleled those of the total CD8 population. There were relatively low numbers of cells that secrete all three cytokines within the CD8^+^ T-cell population after activation with NLV peptide in both age groups; however, there were higher proportions of these cells in the older subjects (*P *< 0·05; Fig.6b). The CMV (NLV)-specific CD8^+^ T cells, regardless of their capacity to secrete cytokines, had shorter telomeres in the old compared with the young subjects (Fig.6c). There were no significant differences between the telomere lengths of the CMV-specific CD8^+^ T cells that secreted all three cytokines compared with the other populations and these cells had an intermediate CD45RA/CD27 defined surface differentiation phenotype compared with those that secreted IL-2 alone or IFN-*γ* and TNF-*α* simultaneously. Collectively our results show that multi-functional CD8^+^ T cells, including those that are specific for CMV (NLV), have an intermediate differentiation phenotype and that their proliferative capacity is unlikely to be restricted by excessive telomere erosion.

## Discussion

The impact of telomere erosion on age-related disease and loss of immune function has been a topic of wide interest. To fully understand the impact of telomere biology on health during aging more accessible and sophisticated methods to assess telomere lengths are required. The current work demonstrates the potential application of multi-coloured flow-FISH to rapidly and reliably determine telomere length, functionality and phenotypic markers within specific cell populations from small cellular samples. Such in-depth telomere analysis may prove a helpful tool in the clinical settings of adoptive cellular immunotherapy, vaccination and diagnostics. The two most compelling correlates for successful responses to adoptive cellular immunotherapy in metastatic cancer trials are often the number of cells transferred and an early state of cellular differentiation.^27^ Indeed, transferring lymphocytes with longer telomere lengths results in enhanced cell survival and improved clinical responses in patients with metastatic melanoma, suggesting that the proliferative potential of the cells is important in improving treatment.^28^,^29^ Leucocyte telomere length has been negatively correlated with increased infection, reduced vaccine responses, cardiovascular and inflammatory disease, and mortality.^8^,^30^–^33^ It is not clear whether the cells with short telomeres exacerbate disease directly or whether telomere shortening in leucocytes is a biomarker for another process such as chronic inflammation; however, the use of the technology we describe will help to unravel these processes.

The importance of a multifunctional T-cell immune response to induce appropriate immunity and clear infections has been described in humans.^1^–^4^ In addition, the capacity of T cells to proliferate upon antigen challenge is also essential to increase the number of antigen-specific cells after immune stimulation, e.g. after vaccination.^34^ However, telomere erosion restricts the proliferative capacity of T cells that are activated repeatedly,^35^–^37^ suggesting that telomere erosion may restrict the persistence of antigen-specific T cells. To date it is not known if the cells that have the shortest telomeres are the ones that are the most multi-functional and this could be one reason why immunity declines during ageing.^38^ In this study we demonstrate that multi-functional IL-2/IFN-*γ*/TNF-*α*-producing cells both within global and a CMV-specific CD8^+^ T-cell populations have intermediate telomere length relative to cells that produce only one or two cytokines after activation, indicating that they are not restricted by telomere erosion. We showed previously that the quality of a response to CMV (NLV epitope) in older subjects may be compromised by the fact that these cells may have lower T-cell receptor avidity^39^ and we are currently investigating the relationship between multifunctional responses, telomere erosion and TCR avidity in individual antigen-specific CD8^+^ T cells.

It is unclear how naive T cells are maintained throughout life because the thymus involutes and thymic output of precursors declines considerably during ageing.^40^–^42^ Our observation that CD45RA^+^ CD27^+^ T cells that are considered to be naive have significantly shorter telomeres in old humans indicates that they have undergone extensive proliferation.^42^ As T-cell receptor activation leads to a loss of CD45RA expression while cytokine-driven homeostatic proliferation does not,^11^,^39^,^43^–^45^ the reduced telomeres in total CD8 as well as CMV (NLV)-specific CD45RA^+^ CD27^+^ CD8^+^ T cells in older humans suggests that proliferation is involved in the homeostatic maintenance of these cells.

An interesting finding is that the CD45RA^+^ CD27^−^ CD8^+^ T cells, that have been shown to have multiple features of a senescent population including restricted proliferative activity^18^,^25^ did not have the shortest telomeres in either the global or CMV (NLV)-specific CD8^+^ T cells. This observation was seen in a Caucasian and an Asian cohort within the CD8^+^ T-cell population defined as CD45RA^+^ CD27^−^, within the CD45RA^+^ CD28^−^ CD8^+^ T-cell population in a Caucasian cohort, and also applies to CD45RA^+^ CD27^−^ populations within the CD4 compartment.^18^ The relative long telomeres of CD45RA^+^ CD27^−^ cells suggest that alternative telomere-independent mechanisms are involved that induce their lack of proliferation and other senescence characteristics. Recent data by our group found that the p38 mitogen-activated protein kinase signalling pathway is highly active in CD45RA^+^ CD27^−^ T cells and this indicates a reversible inhibition of proliferation and telomerase activity in this population.^18^,^25^ This pathway can be triggered by DNA damage, reactive oxygen species, cellular stress and other factors independently of telomere erosion.^41^,^46^ Therefore, although telomere erosion may be a useful biomarker for immune competence and ageing within T cells, other mechanisms are also involved in inducing senescence characteristics that restrict their proliferative lifespan.

In conclusion, we have developed a multi-parameter flow cytometric method that enables the analysis of surface phenotype, cytokine production and telomere length in individual leucocytes. Through standardization with Quantum™ Cy5™ MESF beads, the results from different laboratories using different flow cytometers can be compared directly. This method will facilitate the use of telomere attrition as a biomarker in longitudinal studies and clinical trials. Our results show multiple cytokine secreting cells are at an intermediate stage of differentiation and are not restricted by excessive telomere erosion.

## Abbreviations:

CMV: cytomegalovirus
FISH: fluorescence *in situ* hybridization
IFN: interferon
IL: interleukin
MFI: median fluorescence intensity
PBMC: peripheral blood mononuclear cells
TNF: tumour necrosis factor
TRF: Telomere restriction fragmentation

## Supporting Information

**Table S1.** Results from multiple linear regression fitting age and cytomegalovirus response as covariates for CD8^+^ T-cell subset composition. **Table S2.** Statistical comparisons of percentage of CD8^+^ T-cell cytokine-producing populations following overnight stimulation of peripheral blood mononuclear cells with anti-CD3 and interleukin-2. **Table S3.** Statistical comparisons of telomere length of CD8^+^ T-cell cytokine-producing populations following overnight stimulation of peripheral blood mononuclear cells with anti-CD3 and interleukin-2. **Table S4.** Statistical comparisons of percentage of CD8^+^ T-cell cytokine-producing populations following overnight stimulation of peripheral blood mononuclear cells with NLV peptide. **Table S5.** Statistical comparisons of telomere length of CD8^+^ T-cell cytokine producing populations following overnight stimulation of peripheral blood mononuclear cells with NLV peptide. **Figure S1.** Telomere length of CD8^+^ T-cell subsets defined by CD45RA and CD27 or CD28 expression within European and Asian cohorts.
