# Supplementary material for: Multifunctional cytomegalovirus (CMV)-specific CD8+ T cells are not restricted by telomere-related senescence in young or old adults
Source: Immunology. 2015 Mar 9;144(4):549–60. doi: 10.1111/imm.12409 (PMC4368162; doi:10.1111/imm.12409)

Supplementary table 1. Results from multiple linear regression fitting age and CMV response as covariates for $CD8^+$ T cell subset composition. Table shows the unstandardized coefficient, significance and 95% confidence interval from the output of SPSS software for each CD45RA/CD27 subset. Unit of age is equal to 1 year.

CD45RA$^+$CD27$^+$	**unstandardized coefficient**	***p*-value**	**95.0% CI lower limit**	**upper limit**
**age**	-0.59	<.0001	-0.7	-0.49
**CMV response**	-15.62	<.0001	-21.46	-9.77

CD45RA$^-$ CD27$^+$	**unstandardized coefficient**	***p*-value**	**95.0% CI lower limit**	**upper limit**
**age**	-0.03	0.52	-0.12	0.06
**CMV response**	-6.18	<.01	-10.86	-1.49

CD45RA$^-$ CD27$^-$	**unstandardized coefficient**	***p*-value**	**95.0% CI lower limit**	**upper limit**
**age**	0.125	<0.05	0.005	0.24
**CMV response**	9.05	< 0.01	2.58	15.53

CD45RA$^+$ CD27$^-$	**unstandardized coefficient**	***p*-value**	**95.0% CI lower limit**	**upper limit**
**age**	0.385	<.0001	0.27	0.5
**CMV response**	14.77	< .0001	8.43	21.11

Supplementary table 2. Statistical comparisons of percentage of CD8^{+} T cell cytokine producing populations following overnight stimulation of PBMCs with anti-CD3 and IL-2. Results from Paired T-test.

	IFN	TNF	IL2/IFN	IL2/TNF	IFN/TNF	IL2/IFN/TNF
Young						
IL2	0.0089	0.0013	0.0365	ns	0.0223	ns
IFN	-	0.0124	0.0081	0.0085	0.0491	0.0124
TNF	-	-	0.0012	0.0012	ns	0.0015
IL2/IFN	-	-	-	ns	0.0220	0.0432
IL2/TNF	-	-	-	-	0.0219	ns
IFN/TNF	-	-	-	-	-	0.0229
Old						
IL2	0.0343	0.0001	ns	0.0250	0.0089	ns
IFN	-	ns	0.0252	0.0462	0.0157	ns
TNF	-	-	<0.0001	0.0002	0.0282	0.0002
IL2/IFN	-	-	-	0.0313	0.0081	ns
IL2/TNF	-	-	-	-	0.0096	ns
IFN/TNF	-	-	-	-	-	0.0079

Supplementary table 3. Statistical comparisons of telomere length of CD8^{+} T cell cytokine producing populations following overnight stimulation of PBMCs with anti-CD3 and IL-2. Results from Paired T-test.

	IFN	TNF	IL2/IFN	IL2/TNF	IFN/TNF	IL2/IFN/TNF	Negative
Young							
IL2	0.012	0.004	0.011	ns	0.006	0.018	ns
IFN	-	ns	ns	ns	0.016	ns	.023
TNF	-	-	ns	0.026	ns	0.001	.006
IL2/IFN	-	-	-	ns	ns	ns	ns
IL2/TNF	-	-	-	-	0.028	ns	ns
IFN/TNF	-	-	-	-	-	0.003	.001
IL2/IFN/TNF	-	-	-	-	-	-	.034
Old							
IL2	ns	ns	0.028	0.013	ns	ns	ns
IFN	-	ns	ns	ns	ns	ns	ns
TNF	-	-	ns	ns	ns	ns	ns
IL2/IFN	-	-	-	ns	ns	ns	ns
IL2/TNF	-	-	-	-	ns	0.048	ns
IFN/TNF	-	-	-	-	-	ns	ns
IL2/IFN/TNF	-	-	-	-	-	-	ns

Supplementary table 4. Statistical comparisons of percentage of CD8$^+$ T cell cytokine producing populations following overnight stimulation of PBMCs with NLV peptide. Results from Wilcoxon Signed Rank Test.

	IFN	TNF	IL2/IFN	IL2/TNF	IFN/TNF	IL2/IFN/TNF
Young						
IL2	.043	.176	.018	.028	. 128	.686
IFN	-	.612	.018	.018	.866	.018
TNF	-	-	.018	.018	.612	.018
IL2/IFN	-	-	-	.237	.028	.043
IL2/TNF	-	-	-	-	.018	.018
IFN/TNF	-	-	-	-	-	.018
Old						
IL2	.138	.080	.225	.893	.043	.176
IFN	-	.345	.043	.043	.345	.043
TNF	-	-	.043	.043	.225	.043
IL2/IFN	-	-	-	.893	.043	.866
IL2/TNF	-	-	-	-	.043	.500
IFN/TNF	-	-	-	-	-	.043

Supplementary table 5. Statistical comparisons of telomere length of CD8$^+$ T cell cytokine producing populations following overnight stimulation of PBMCs with NLV peptide. Results from Wilcoxon Signed Rank Test.

	IFN	TNF	IL2/IFN	IL2/TNF	IFN/TNF	IL2/IFN/TNF	Negative
Young							
IL2	ns	.028	.043	ns	.028	ns	ns
IFN	-	.028	ns	ns	.028	ns	ns
TNF	-	-	ns	ns	ns	ns	.028
IL2/IFN	-	-	-	ns	ns	ns	ns
IL2/TNF	-	-	-	-	ns	ns	ns
IFN/TNF	-	-	-	-	-	ns	.028
IL2/IFN/TNF	-	-	-	-	-	-	ns
Old							
IL2	ns	ns	ns	ns	ns	ns	ns
IFN	-	ns	ns	ns	.043	ns	.043
TNF	-	-	ns	ns	ns	ns	.043
IL2/IFN	-	-	-	ns	ns	ns	ns
IL2/TNF	-	-	-	-	ns	ns	-
IFN/TNF	-	-	-	-	-	ns	.043
IL2/IFN/TNF	-	-	-	-	-	-	ns

Supplementary Figure 1

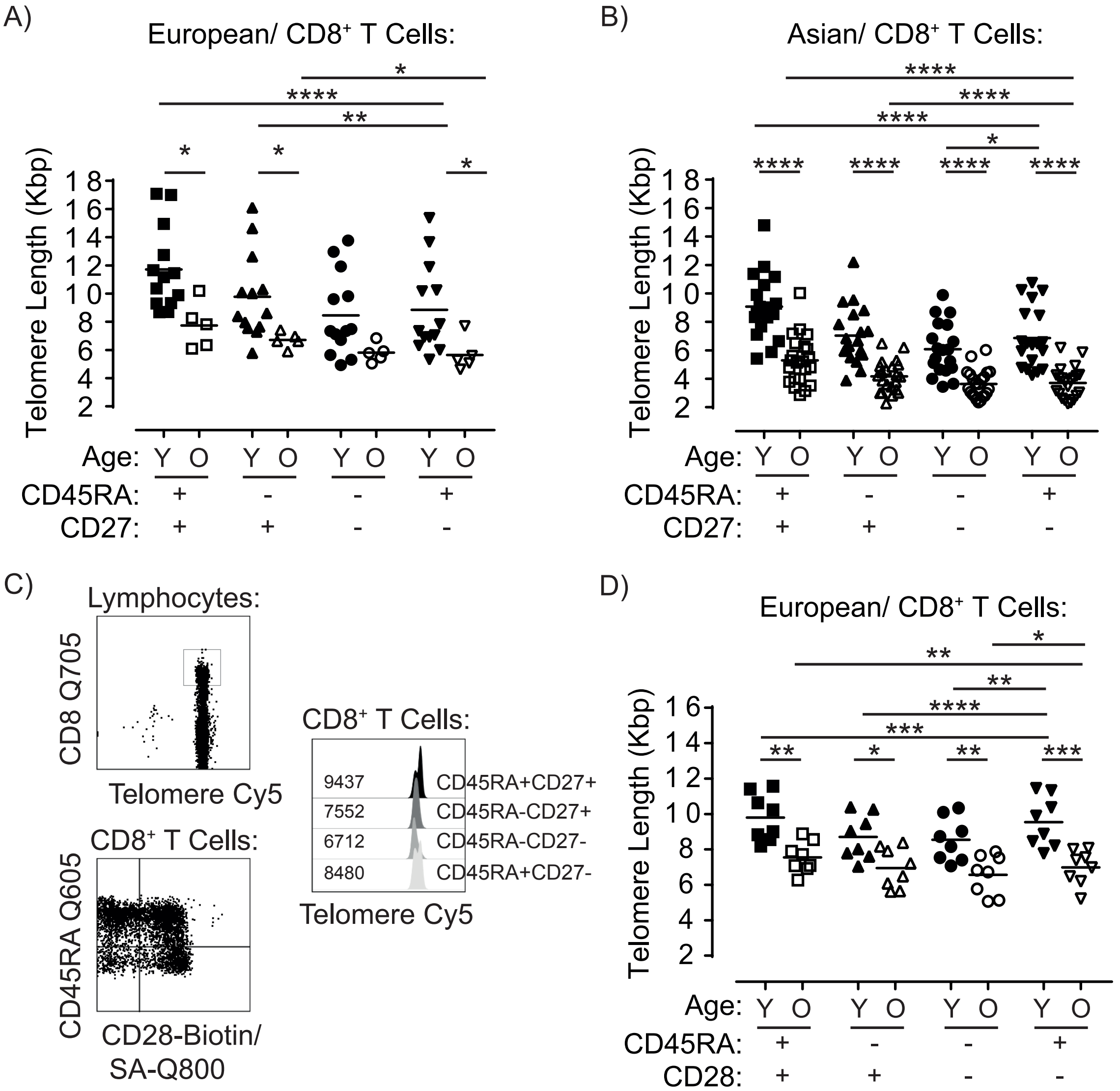

Supplement: Supplementary file 1 [file imm0144-0549-sd1.pdf]
